# Regulation of Calcitriol Biosynthesis and Activity: Focus on Gestational Vitamin D Deficiency and Adverse Pregnancy Outcomes

**DOI:** 10.3390/nu7010443

**Published:** 2015-01-09

**Authors:** Andrea Olmos-Ortiz, Euclides Avila, Marta Durand-Carbajal, Lorenza Díaz

**Affiliations:** Department of Reproductive Biology, National Institute of Medical Sciences and Nutrition Salvador Zubirán, Vasco de Quiroga No. 15, Tlalpan 14000, México City, Mexico; E-Mails: nut.aolmos@gmail.com (A.O.-O.); euclides.avilac@incmnsz.mx (E.A.); mdurand65@gmail.com (M.D.-C.)

**Keywords:** micronutrient, placenta, maternal diet, gestational pathologies

## Abstract

Vitamin D has garnered a great deal of attention in recent years due to a global prevalence of vitamin D deficiency associated with an increased risk of a variety of human diseases. Specifically, hypovitaminosis D in pregnant women is highly common and has important implications for the mother and lifelong health of the child, since it has been linked to maternal and child infections, small-for-gestational age, preterm delivery, preeclampsia, gestational diabetes, as well as imprinting on the infant for life chronic diseases. Therefore, factors that regulate vitamin D metabolism are of main importance, especially during pregnancy. The hormonal form and most active metabolite of vitamin D is calcitriol. This hormone mediates its biological effects through a specific nuclear receptor, which is found in many tissues including the placenta. Calcitriol synthesis and degradation depend on the expression and activity of CYP27B1 and CYP24A1 cytochromes, respectively, for which regulation is tissue specific. Among the factors that modify these cytochromes expression and/or activity are calcitriol itself, parathyroid hormone, fibroblast growth factor 23, cytokines, calcium and phosphate. This review provides a current overview on the regulation of vitamin D metabolism, focusing on vitamin D deficiency during gestation and its impact on pregnancy outcomes.

## 1. Vitamin D Synthesis and Metabolism

UVB radiation from sunlight initiates vitamin D (VD) biosynthesis in the skin by bioconverting 7-dehydrocholesterol to previtamin D_3_, which is thermally isomerized to VD_3_. VD may also be obtained from the diet to a lesser extent. The nutritional forms of VD include both VD_3_ (cholecalciferol) from animal origin and VD_2_ (ergocalciferol) from fungi and plant origin. Once formed in the skin or absorbed in the intestine, VD is released into the circulation and transported by the VD-binding protein (DBP) to the liver, where it is converted by the VD-25-hydroxylase (CYP2R1) into 25-hydroxyvitamin D (calcidiol, 25OHD). Calcidiol is the major vitamin D metabolite present in the circulation and the best indicator of VD nutritional status. Nevertheless, this metabolite is not the active form of VD, and therefore needs further activation by a second hydroxylation step catalyzed by the enzyme 25OHD-1-α-hydroxylase (CYP27B1) in order to generate 1,25-(OH)_2_D_3_ (calcitriol), which is the hormonal form and most active VD metabolite. CYP27B1 is primarily expressed in the kidney but also may be found in different tissues including the placenta. The bioavailability of calcitriol is tightly regulated to restrict the biological actions of this hormone in target cells while maintaining calcium and phosphate homeostasis, and the enzyme in charge of degrading calcitriol to water-soluble and less active metabolites is the 1,25-(OH)_2_D_3_-24-hydroxylase (CYP24A1), which in turn is highly upregulated by calcitriol itself, as a negative feedback mechanism [1,2].

Importantly, in order for CYP27B1 to fulfill its duty of producing calcitriol, there must be enough substrate available. This requirement is readily accomplished by the endocytic receptors megalin/cubilin and the adaptor protein disabled-2 (Dab2) present in the kidney, where 25OHD in complex with DBP is filtered through the glomerulus and reabsorbed in the proximal tubules by the cooperative action of these proteins [3,4,5]. Inside the cell, DBP is degraded and 25OHD is released and hydroxylated by CYP27B1. This specialized mechanism allowing renal uptake and activation of 25OHD is paramount for calcitriol production, as demonstrated by Nykjaer A. *et al.* [3] in rats infused with ^3^H-25OHD/DBP and where megalin activity was blocked. In these animals, no conversion products (*i.e.*, ^3^H-calcitriol) were recovered from serum samples. In contrast, in animals with intact and active megalin that were also infused, ^3^H-calcitriol was readily detected in their plasma samples. Similarly, the importance of cubilin in the 25OHD reabsorption process is evident in human patients carrying inactivating mutations in cubilin gene (Imerslund–Gräsbeck disease), which exhibit abnormal urinary excretion of 25OHD and DBP [4].

## 2. Calcitriol Biological Effects

Calcitriol actions are mediated through the vitamin D receptor (VDR), a high-affinity ligand-activated transcription factor. Once bound to its ligand, the VDR heterodimerizes with the retinoid X receptor (RXR). This complex recognizes vitamin D response elements (VDRE) in the promoter regions of VD target genes and recruits co-activators or co-repressors in order to induce or repress gene transcription [6]. In addition, non-genomic calcitriol-dependent biological effects can also take place in cells, involving second messengers generated by membrane-initiated signaling pathways [7,8]. Indeed, the classic VDR and the membrane-associated rapid response steroid-binding protein (MARRS) found in the cell membrane may bind calcitriol and initiate the activation of numerous pathways involving protein kinase C (PKC) [9], mitogen-activated protein kinase (MAPK) [10], protein kinase A (PKA) [11,12], phosphatidyl inositol phosphate [13] and Ca^2+^ and chloride channels [8,14]. Very important processes are mediated through these rapid signaling cascades, such as insulin secretion and transcaltachia [15,16,17]. In general, the known VDRE-containing genes can be grouped in very diverse biological networks including bone and mineral metabolism (*i.e.*, osteopontin [18]); cell life and death (comprising proliferation, differentiation, migration and apoptosis, *i.e.*, p21 [19]), metabolism and cardiovascular health (*i.e.*, cystathionine β-synthase [20], renin [21] and VEGF [22]), immune function (*i.e.*, cathelicidin hCTD, LL-37 [23,24]) and detoxification (*i.e.*, CYP3A4, CYP24A1 [25]).

## 3. Regulation of the VD-Metabolizing Enzymes

Renal CYP27B1, which is responsible for the most part of circulating calcitriol, is mainly regulated by parathyroid hormone (PTH) -as a signal of calcium status-, fibroblast growth factor 23 (FGF23) -as a signal of serum phosphate levels- and calcitriol itself -as a negative feedback regulatory loop- [26]. When serum calcium levels are low, PTH is secreted by the parathyroid gland in order to stimulate renal CYP27B1 by a cyclic AMP (cAMP)-dependent mechanism [27]. PTH also causes CYP24A1 mRNA degradation in the kidney [28]. Calcitriol production is then boosted, thereby increasing blood calcium levels by promoting absorption of dietary calcium from the gastrointestinal tract, increasing renal tubular calcium reabsorption and stimulating the release of calcium from bone. As feedback effects, the synthesis and secretion of PTH are then inhibited by calcitriol and FGF23, the latter being produced in the bone [29]. FGF23 also potently inhibits CYP27B1 in response to elevated phosphate levels [30]. Finally, calcitriol inhibits its own production by different mechanisms comprising PTH inhibition, direct transcriptional repression of the CYP27B1 gene, and FGF23 and CYP24A1 induction [31]. Other factors that stimulate renal CYP27B1 activity and/or expression are insulin-like growth factor type I (IGF-I) and calcitonin [32,33,34].

## 4. Extra-Renal Regulation of Vitamin D-Hydroxylases

Extra-renal CYP27B1 is regulated in different ways than those observed in the kidney, in a tissue-specific manner. For instance, macrophage and monocyte CYP27B1 is not stimulated by PTH but rather by cytokines such as interferon-γ (INF-γ) and tumor necrosis factor-α (TNF-α) [35,36,37,38]. In addition, CYPB7B1 in immune cells is not readily negatively regulated by calcitriol. Likewise, the negative regulatory loop exerted by CYP24A1 is not very effective; therefore, 25OHD availability is the limiting factor for calcitriol synthesis in these cells [38]. In a similar manner as in immune cells, in keratinocytes, TNF-α and INF-γ potently induce CYP27B1 whereas calcitriol does not directly inhibit this gene [39,40,41]. Nevertheless, CYP24A1 is induced by calcitriol and efficiently degrades bioactive calcitriol, thus regulating calcitriol availability in the epidermis [39]. The transcriptional induction of CYP24A1 by calcitriol is feasible considering the existence of multiple VDREs within its promoter. However, other regulatory mechanisms can also play a role in CYP24A1 regulation, such as alternative splicing, epigenetic gene silencing and metabolism of its mRNA [42,43,44,45].

In a comparable fashion as immune cells and keratinocytes, proinflammatory cytokines induce CYP27B1 gene expression in the human placenta [46]. Interestingly, in this tissue, CYP24A1 expression is also stimulated by these factors, suggesting that both synthesis and catabolism of placental calcitriol are locally affected by inflammatory cytokines [46]. Nevertheless, we have shown that TNF-α increased significantly the expression of CYP24A1 over CYP27B1, whereas IFN-γ preferentially stimulated CYP27B1. These observations, together with those showing that cultured trophoblasts secrete significantly more TNF-α than INF-γ, suggest that in the placenta, increased TNF-α secretion may limit calcitriol bioavailability [46]. The latter agrees with *in vivo* evidence showing low serum calcitriol and low placental CYP27B1 expression and calcitriol production in preeclampsia (PE), a pregnancy-associated condition characterized by an exacerbated pro-inflammatory profile.

In contrast to immune and epidermal cells, calcitriol in the placenta is able to transcriptionally inhibit CYP27B1 expression, which is mediated by both the VDR and a cAMP-dependent mechanism [12]. In this tissue, CYP24A1 gene expression is also potently induced by calcitriol [12]. However, it is also recognized that the CYP24A1 gene is methylated in the human placenta, suggesting that epigenetic decoupling of VD feedback catabolism may play an important role in maximizing calcitriol bioavailability at the feto-maternal interface [43]. Nevertheless, as discussed previously by Rosen *et al.* [47], this assumption is not supported by functional studies, since there is unequivocal evidence for human placental synthesis of 24,25-dihydroxyvitamin D_3_, the main metabolic product of CYP24A1 [48]. Moreover, in this seminal article by Rubin and colleagues, under physiologic concentrations of 25OHD, placental trophoblasts preferentially synthesize 24,25-dihydroxyvitamin D_3_ over calcitriol [48], which may explain why fetal levels of 24-hydroxylated VD metabolites are 40-fold higher than calcitriol [47,49,50]. These studies strongly suggested that placental CYP24A1 expression and activity, together with substrate availability are important limiting factors for calcitriol synthesis in the feto-maternal interface. Other hormonal factors affecting placental VD-metabolism are IGF-I and the natural occurring calciotropic hormones calcitonin and PTH [12,51]. IGF-I stimulates biotransformation of 25OHD into calcitriol [51], while both calcitonin and PTH regulate the placental VD-hydroxylases in the same manner as calcitriol and cAMP, inducing CYP24A1 while repressing CYP27B1 gene expression, thereby favoring VD catabolism [12].

## 5. Physiological Changes of 25OHD, Calcitriol, DBP and VDR during Pregnancy

During pregnancy, significant changes in maternal serum calcitriol, DBP and placental VDR take place and interact to acquire extra calcium for adequate fetal bone mineralization (for a recent review see Brannon PM and Picciano MF [52]). Indeed, the fetus may accumulate up to 30 g of calcium at term, and to satisfy this demand, VD metabolism is boosted in order to increase calcium intestinal absorption [53]. Collectively, these changes include increased maternal serum calcitriol, DBP, placental VDR and renal and placental CYP27B1 activity, without changes in serum 25OHD or calcium levels. In fact, maternal ionized calcium does not increase despite higher circulating calcitriol [54,55,56,57]; instead, serum calcium remains normal as calcium is transferred to the fetus. This equilibrium could be attributed to the concomitant normal rise in calciuria [55,56,57,58,59], precluding the risk of hypercalcemia. Fetal serum calcium levels are higher than those observed in the mother, a situation that requires specific mechanisms for calcium transport against a concentration gradient. In this regard, the active transplacental passage of calcium to the fetus is mediated by placental expression of calcium binding proteins such as calbindin-D9k and 28k [60,61,62]. Interestingly, placental VDR has shown to be a positive predictor of fetal femur length and is positively correlated with maternal-to-fetal transfer of calcium [63], suggesting that fetal skeletal growth could be affected by VDR-dependent mechanisms; and therefore, the relative VDR placental abundance would be a preponderant feature for fetal bone health.

Maternal 25OHD serum levels remain constant across pregnancy [56,57,59,64], suggesting that the increment observed in serum calcitriol levels is independent of changes in its precursor synthesis. Maternal 25OHD crosses the placental barrier and represents the main pool of VD in the fetus. In fact, serum fetal (cord blood) 25OHD levels are on average 25% reduced compared to maternal serum and correlate well with mother 25OHD levels [65,66]; therefore, VD deficiency in the mother could be vertically transmitted to the fetus.

During pregnancy, serum calcitriol rises from the first trimester, doubling its concentration compared to non-gravid women by the end of the third trimester and returning to normal values after delivery [54,56,58,59,64,67]. This physiological rise in calcitriol levels observed during pregnancy could be related to increased synthesis rather than decreased clearance [68]. Increased synthesis of calcitriol is linked to higher CYP27B1 activity in maternal kidney, placental trophoblasts and decidua [69,70,71].

The mechanisms underlying improved CYP27B1 activity during pregnancy remain elusive, partly because its known regulatory factors stay unchanged during this period, such as PTH [54,55,56,59], which may be even lower with respect to non-gravid women [58,64]. Also, a murine PTH null model supports that PTH does not contribute to increased calcitriol levels during pregnancy [72]. It has been hypothesized [64,73] that a potential regulatory factor for CYP27B1 could be the PTH analog PTH-related peptide (PTH-rP), which is synthesized by fetal parathyroids and placenta [74] and increases throughout pregnancy. PTH-rP could reach the maternal circulation and, after binding to PTH type 1 receptor (PTHR1), it may induce gene expression of CYP27B1 in the kidney and catalyze calcitriol synthesis for endocrine actions. However, this mechanism could not completely explain the higher activity of CYP27B1 in placenta because, as previously described, this tissue has its own mechanisms governing VD metabolism. In fact, PTH downregulates placental CYP27B1 gene expression [12].

The placental contribution to maternal calcitriol levels was demonstrated in 1978 by Weisman and colleagues in a nephrectomized pregnant rat model. In that study, the authors showed that anephric rats can synthesize calcitriol and 24,25-dihydroxyvitamin D_3_ from 25OHD and that the fetoplacental unit is the most likely site of production of such metabolites [75]. Later, the *in vitro* synthesis of 24,25-dihydroxyvitamin D_3_ and calcitriol by the human placenta was demonstrated [69]. Interestingly, in a model of chronic renal failure by Blum and colleagues [76], nephrectomized rats had lower calcitriol levels in comparison with normal rats, but during pregnancy the nephrectomized group reached similar levels to those observed in pregnant controls, despite kidney absence, which emphasized the important contribution of the placenta [76]. In 2000, studies in cultured human placental syncytiotrophoblasts showed that the synthesis of calcitriol from its endogenous precursor was driven by an enzymatic 1α-hydroxylation mechanism, since these cells expressed a CYP27B1 gene transcription product with a nucleotide sequence identical to that of transcripts previously characterized in the human kidney [70]. Later, the CYP27B1 protein localization was demonstrated in human placental decidual and trophoblastic cells [77].

In relation to VDR, it has been shown that its gene and protein expression is higher in placental and decidual tissue during the first and second trimesters in comparison with term placentas [78,79], in a similar manner as CYP27B1, with highest levels of expression occurring in first trimester decidua [78], thus suggesting a more preponderant role for calcitriol during the first part of pregnancy. It is likely that this is related to the importance of maintaining an anti-inflammatory setting for the acceptance of the fetal allograft. This is discussed further in Section 6.

Regarding DBP, two longitudinal studies indicate that this protein increases 25% to 56% during pregnancy [59,67], but the mechanisms leading to this increment are still unknown. It has been hypothesized that the DBP rise may be due to increased calcitriol concentration in gestation. In support of this assumption, serum DBP levels in pregnant women correlated with serum calcitriol levels [80].

The rise in DBP during pregnancy is intriguing and should be taken into consideration for the analysis of VD homeodynamics and physiological impact during this period. Actually, a great debate has been taking place regarding which form of 25OHD is the more suitable for activation, the “free” or the DBP-bound form. In this regard, the “free hormone” hypothesis sustains that the free steroid, by diffusing freely through the cell membrane, is the one available for activation and thereafter capable of performing biological effects, while the hormone bound to its carrier protein is considered to be sequestered and therefore, not bioavailable. However, this hypothesis has been confronted in the last years, given the important transporting and interacting mechanisms existing between the megalin/cubilin complex and DBP/25OHD, together with the observed disparity between the expected amounts of free hormone available for passive diffusion and the levels required to efficiently occupy intracellular target receptors. For an excellent review see: Chun *et al.* [81]. Interestingly, Chun and colleagues have recently proposed a viable hypothesis considering a role for DBP in tissue discrimination of 25OHD_2_ and 25OHD_3_. Given that 25OHD_2_ binds to DBP with lower affinity than 25OHD_3_, the kidney would preferentially use the latter metabolite, while cells in the immune system might profit of a greater pool of 25OHD_2_ for antimicrobial peptide induction [81].

It is noteworthy mentioning that megalin/cubilin-mediated 25OHD/DBP endocytosis may also take place in the placenta, since these receptors are expressed in this tissue [82,83,84] and placental calcitriol production has been proved [69,75], suggesting that circulating 25OHD is accessible to placental CYP27B1 by active transport. However, trophoblastic production of calcitriol may also be explained by the steroid freely diffusing through the cell membrane, and consequently both free and bound-25OHD might represent a bioavailable material for placental CYP27B1. Therefore, even if the uptake of 25OHD by proximal tubule cells evidently depends on the internalization of DBP, the functional significance of the placental megalin/cubilin endocitic complex expression and functionality remains far from clear.

Interestingly, mice with genetic ablation of the DBP gene only presented classic VD-associated problems when maintained under a low VD diet, supporting a role for DBP in maintaining stable serum stores of VD metabolites while modulating the rates of its bioavailability, activation, and end-organ responsiveness [85].

## 6. Calcitriol Effects during Pregnancy

One of the main activities attributed to calcitriol during pregnancy is to increase calcium absorption and to upregulate placental calcium transport. However, since VDR and CYP27B1 are also expressed in reproductive feminine tissues like the uterus, ovary, endometrium, fallopian epithelial cells and placenta [86,87,88], other potential paracrine and autocrine actions of calcitriol cannot be discarded. In this regard, during gestation, VD-dependent regulation of the immune function is paramount, since an adequate balance of the cytokine profile is necessary for pregnancy success. Specifically, calcitriol plays a dual role aimed at improving the innate immune response while restraining exacerbated inflammation. Calcitriol achieves this by inhibiting pro-inflammatory cytokines such as TNF-α, IFN-γ and interleukin-6 (IL-6), while at the same time induces the potent antimicrobial peptide hCTD in the fetoplacental unit [89,90,91,92]. In this tissue, calcitriol also stimulates other antimicrobial peptides known as β-defensins (HBDs), in particular HBD2 and HBD3 [93].

Calcitriol biological effects in the placenta are also related to hormonogenesis and overall placental physiology. In particular, calcitriol induces endometrial decidualization and estradiol and progesterone synthesis, but also regulates the expression of human chorionic gonadotropin and placental lactogen expression [94,95,96,97]. Table 1 lists currently known targets regulated by calcitriol in human placenta.

Considering these important effects of calcitriol on human pregnancy, it is not surprising that VD inadequacy may contribute to many gestation-associated disorders. Herein, we revise some common adverse pregnancy outcomes associated with maternal VD deficiency.

## 7. Vitamin D Deficiency and Adverse Pregnancy Outcomes

As previously mentioned, circulating 25OHD represents the best indicator of VD status. This metabolite was eligible instead of calcitriol for two principal reasons [98]: (1) 25OHD is present in a higher concentration than calcitriol (ng/mL *vs.* pg/mL); and (2) in a deficient 25OHD scenario, PTH is stimulated and consequently induces renal CYP27B1 expression and, therefore, calcitriol synthesis. Consequently, this could derivate in a relative and transient “normality” in calcitriol levels, given its short half-life (few hours for calcitriol instead of 2–3 weeks for 25OHD [99,100]).

The spectrum of VD status has been established considering the known risk for adverse consequences [101]. 25OHD values lesser than 25 nmol/L are related to rickets and osteomalacia and therefore are labeled as “severe deficiency”. 25OHD values lesser than 50 nmol/L may sustain long-term adverse health consequences and are classified as “deficiency.” The 75 nmol/L cut-off is the point above which there is no upper stimulation of PTH [102] and was thereafter designated as “sufficient.” However, Heaney pointed out that 25OHD concentrations < 80 nmol/L are associated with reduced calcium absorption and osteoporosis risk [103]; therefore, values lesser than 75 nmol/L are considered as “insufficient.” The conversion factor between 25OHD nmol/L and ng/mL is 2.5 (Table 2).

**Table 1:** Targets modulated by calcitriol in the human placenta (+ = Stimulation; − = Inhibition).

Target	Biological Network	Regulation	Bioeffect	Cell Type	Reference
Placental lactogen	Cell life and death	+	Growth control	Trophoblast	[97]
TNF-α	Immune function	−	Restraining inflammation	Trophoblast	[91,92]
		−	Immunosupression	Decidual cells	[89]
IL-6	Immune function	−	Restraining inflammation	Trophoblast	[91,92]
		−	Immunosupression	Decidual cells	[89]
CSF2 (colony stimulating factor 2)	Immune function	−	Immunosupression	Decidual cells	[89]
hCTD	Immune function	+	Restraining infection	Trophoblast	[90]
				Decidual cells	[89]
CYP24A1	Bone and mineral metabolism	+	Calcitriol catabolism	Trophoblast	[12]
				Decidual cells	[104]
CYP27B1	Bone and mineral metabolism	−	Calcitriol synthesis	Trophoblast	[12]
IL-10 (Interleukin 10)	Immune function	−	Reducing risk of infection	Trophoblast	[105]
KCNH1 (Potassium voltage-gated channel)	Cell life and death	−	Unknown	Trophoblast	[106]
Calbindin-D 28 kDa	Bone and mineral metabolism	+	Calcium transfer	Trophoblast	[61,62]
Calbindin-D 9 kDa	Bone and mineral metabolism	+	Calcium transfer	Trophoblast	[61]
hCG (human chorionic gonadotrophin)	Cell life and death	+, −	Maintenance of pregnancy	Trophoblast	[96]
3β-HSD (3β-hydroxysteroid dehydrogenase) *	Cell life and death	+	Progesterone synthesis	Trophoblast	[95]
CYP19 (aromatase) *	Cell life and death	+	Estradiol synthesis	Trophoblast	[95]
Prolactin	Cell life and death	+	Establishment of pregnancy	Decidual cells	[107]
VDR	Bone and mineral metabolism	+	Allowing calcitriol actions	Trophoblast	[62]
Platelet-activating factor acetylhydrolase	Cell life and death	−	Inactivation of platelet-activating factor	Decidual macrophages	[108]
HOXA10 (Homeobox A10)	Cell life and death	+	Embryo implantation	Decidual cells	[109]
Osteopontin	Bone and mineral metabolism	+	Embryo implantation	Decidual cells	[104]
HBD2	Immune function	+	Restraining infection	Trophoblast	[93]
HBD3	Immune function	+	Restraining infection	Trophoblast	[93]

* Only observed at the enzyme activity level.

**Table 2:** Cut-offs in vitamin D status according to the Endocrine Society [110].

Vitamin D Status	25OHD (nmol/L)	25OHD (ng/mL)
Severe deficiency	<25	<10
Deficiency	<50	<20
Insufficiency	<75	<30
Sufficiency	75–110	30–44
Toxicity	>250	>100

Table 2 shows the criteria for VD status as proposed by the Endocrine Society. We believe this criteria is more applicable to this review since it provides guidance for clinicians caring for patients, as compared to the reference proposed by the Institute of Medicine, more likely intended for normal healthy populations only to ensure skeletal health [111].

Observational studies have described an association between insufficiency or deficiency in 25OHD levels and adverse pregnancy and neonatal outcomes including PE, gestational diabetes, bacterial vaginosis, recurrent abortion, premature rupture of membranes (PROM), preterm delivery, cesarean section, intrauterine growth restriction and also impaired fertility treatment.

The VD literature is growing rapidly and there are some recent and detailed reviews and meta-analyses about adverse pregnancy outcomes and VD [52,112,113,114,115,116,117,118,119]. The purpose of this section is therefore to show a broad overview of VD status and its relationship with epidemiological and observational data in adverse pregnancy outcomes.

### 7.1. VD and Preeclampsia (PE)

PE is a hypertensive disease associated to gestation. It is clinically diagnosed by new-onset hypertension (140/90 mmHg) and proteinuria (300 mg/24 h or protein dipstick 1+ or greater) after the 20th gestational week [120,121]. The major cause related to this disease is abnormal placentation secondary to insufficient throphoblastic invasion that disrupts the endocrine, immunologic and angiogenic environment, resulting in the clinical manifestation of PE [122,123]. Interestingly, a multicenter study with 2030 pregnant women of an American cohort from 1959 to 1966 showed that higher maternal circulating 25OHD levels were associated with a significantly lower risk of placental vascular pathology (hemorrhage, infarcts, microinfarcts, decidual atheromas or thrombosis of cord vessels) in pregnant women carrying male fetuses [124]. From this perspective, VD could be a protective factor for the correct development of placental vasculature.

There is a general consensus in the literature about preeclamptic women having lower 25OHD and calcitriol serum levels compared to normotensive normoevolutive pregnant women [125,126,127,128,129]. Indeed, VD deficiency is more common among preeclamptic women [130,131,132,133,134,135]. This could partially be explained by significantly lower CYP27B1 placental expression and therefore, lower calcitriol biosynthesis in preeclamptic *versus* normal placentas [136].

The relationship between VD, especially 25OHD serum levels, and the risk of PE development has been extensively analyzed in the medical literature. Herein, we resume the studies that positively correlate VD deficiency and PE risk (Table 3).

**Table 3:** Observational studies on 25OHD serum levels and the risk for PE development.

Reference	Sample Size (PE Cases *vs.* Controls)	Weeks of Gestation for Blood Sampling	25OHD Cut off	Risk for PE Development (OR (95% CI))
[130]	100 PE and 100 controls	>24	<75 nmol/L	3.26 (1.12–9.54)
			<37.5 nmol/L	4.23 (1.4–12.8)
[131]	33 PE, 79 eclamptic and 76 control	≥ 20 weeks, prior to magnesium sulfate therapy	<5 nmol/L	3.9 (1.18–12.87) for PE
				5.14 (1.98–13.37) for eclampsia
[132]	32 PE and 665 controls	24–26	<50 nmol/L	3.24 (1.37–7.69)
[133]	51 severe PE and 204 controls	15–20	<50 nmol/L	3.63 (1.52–8.65)
[134]	55 PE and 219 controls	22	<37.5 nmol/L	5.0 (1.7–14.1)

PE = Preeclampsia; OR = odds ratio; CI = confidence interval.

Interestingly, Robinson and colleagues [129] found that a 10 ng/mL increase in 25OHD levels yields a 63% decrease in the risk of severe PE, strongly suggesting that pregnant women should have VD sufficiency in order to lower the risk for PE development. In support of this postulation, recently, Bodnar and coworkers [137] studied a large cohort with 717 PE women and 2,986 control women, concluding that maternal VD deficiency is a clear risk factor for severe PE development. Specifically, they found that maternal 25OHD levels ≥ 50 nmol/L reduce in 40% the risk of severe PE development in comparison to women with 25OHD < 30 nmol/L. Another study reports that both 25OHD and soluble VEGF receptor type 1/placental growth factor (sFlt-1/PlGF) ratio at 15–20 weeks of gestation were significant predictors of severe PE [138]. Similarly, a Norwegian study [139] revealed that increased VD intake (15–20 μg/day) decreases about 25% the risk for PE development, and four independent meta-analyses showed a significant association between PE and 25OHD insufficiency or deficiency compared with control groups [112,119,140,141].

Despite all these reports, other authors inform they did not find any relation between maternal 25OHD levels and risk of PE development [142,143].

In summary, this data supports that maintaining VD sufficiency is a relatively simple measure (by cholecalciferol supplementation or reasonable skin sun exposure) for preventing one of the major causes in mother and baby morbidity-mortality, namely, preeclampsia.

### 7.2. VD and Bacterial Vaginosis

Bacterial vaginosis (BV) is a common infectious disease in reproductive-aged women. It is caused by the replacement of normal vaginal flora (especially Lactobacillus) for mixed anaerobic bacteria [144]. Its importance during pregnancy resides in the association of BV with adverse gynecologic and obstetric outcomes such as PROM, which can cause spontaneous or induced preterm delivery [145].

Interestingly, the National Health and Nutrition Examination Survey (NHANES) 2001–2004 reported that black women suffer two-fold more BV cases in comparison to white women (51.6% *vs.* 23.2%, respectively) [146]. This proportion could be explained by the fact that high melanin levels in darkly pigmented skin blocks ultraviolet radiation reducing cutaneous VD photosynthesis and consequently, decreasing the well-known antimicrobial activity of endogenous calcitriol [147,148]. Observational studies support this hypothesis. A prospective cohort study [149] developed at Pittsburgh University followed 469 pregnant women from <16th gestational week to term. It was observed that mothers with 25OHD serum levels <20 nmol/L had a 65% increased risk of developing BV compared to 25OHD sufficient women (>80 nmol/L), and similarly, in a subsample of the Nashville Birth Cohort [150], 25OHD serum levels were lower in women who developed BV during pregnancy. In a secondary analysis of data from the NHANES 2001–2004, Hensel and coworkers [151] described that VD insufficiency or deficiency had a statistically significant association with BV only among pregnant women (adjusted OR 2.87, 95% CI 1.13–7.28). Interestingly, a meta-analysis comprising 16 independent studies showed that women with bacterial or viral infections presented 2.1 increased risk (95% CI: 1.6–2.7) of PE development [152], suggesting a probable common risk factor which could be VD deficiency.

Besides BV, calcitriol can prevent other kinds of infections during pregnancy. In human bladder biopsies and established bladder cell lines, 25OHD treatment induced hCTD gene expression, which diminished uropathogenic *E. coli* infection, suggesting that adequate 25OHD serum levels could help prevent urinary tract infections [153].

In addition, a case-control study [154] reports that pregnant women with 25OHD insufficiency at 14–16 gestational week had 2.1-fold increased risk in developing severe to moderate periodontal disease (95% CI: 0.99–4.5).

The physiological mechanisms underlying these observations are possibly related to immune responses regulated by calcitriol. In fact, as previously mentioned, calcitriol can induce innate immune responses by activating hCTD in placenta, macrophages and dendritic cells [90,155]. The antimicrobial hCTD is an active peptide with broad spectrum antimicrobial activity (anti-Gram-positive and Gram-negative bacteria, mycobacterias, spirochetas and yeasts). Its mechanism of action includes bacterial membrane disruption and activation of toll-like receptors and macrophage and neutrophil chemotaxis [156]. Recently, it was also found that calcitriol can induce hCTD expression, multivesicular endosomes and phagolysosome biogenesis in macrophages, leading to microbial killing through autophagy [157,158,159]. Other important antimicrobial peptides induced by calcitriol in the placenta are HBD2 and HBD3, which also play a shielding role upon infections. Conjointly, these endogenous antibiotics provide an efficient mechanism of front-line defense since they have the capacity to kill a wide variety of microorganisms throughout the female reproductive tract [160]. Interestingly, it has been shown that IL-10, a physiological suppressor of maternal active immunity, downregulates placental antimicrobial peptides expression, which may be permissive for microbial invasion, since the placenta represents a mechanical and immunological barrier essential to restrict infection progress. However, calcitriol is able to antagonize IL-10 suppressive effects upon placental innate defenses by downregulating IL-10 expression, while at the same time restraining exacerbated inflammation and subsequently helping pregnancy to continue in quiescence [93,105]. These data indicate that adequate VD levels are crucial to enhance immunity.

By reducing infections, calcitriol may exert protective effects upon PROM. However, another mechanism might be involved in PROM prevention: calcitriol, either alone or combined with lipopolysaccharide endotoxin, decreases oxitocin and connexin 43 expression in myometrial smooth muscle cells, both proteins are associated with uterine contractions [161]. These results suggest that calcitriol can modulate uterine quiescence even under bacterial infection and therefore can prevent the abnormal uterine contractions that favor PROM and preterm delivery. In this sense, 25OHD could exert a protective role in preterm birth but this still remains unclear, since in a multicenter American cohort with twin-gestation women [162], it was observed that women with sufficiency in 25OHD (>75 nmol/L) had a 60% lower risk of preterm labor in comparison to those <75 nmol/L (OR 0.4, 95% CI 0.2–0.8). However, in a case-control study with women at high risk for prior preterm birth, VD status at 16–22 gestational weeks was not associated with recurrent preterm birth [163].

### 7.3. Vitamin D and Gestational Diabetes

Gestational diabetes mellitus (GDM) is defined as carbohydrate intolerance resulting in hyperglycemia of variable severity with new-onset or first recognition during pregnancy [164,165]. GDM, one of the most common complications of pregnancy, is related to adverse outcomes that increase morbidity and mortality in mothers and neonates, including hypertension, PE, urinary tract infection, caesarean delivery, fetal macrosomia, neonatal hypoglycemia and high long-term risk for metabolic syndrome or diabetes mellitus type 2 (DM2) development [165,166,167].

The classic risk factors known for GDM include maternal overweight status or obesity, prior history of GDM, family history of DM2, antecedent of macrosomic infant, and increased maternal age [165]. However, since 1980 [168], there is a constantly growing body of evidence that supports a connection between VD and insulin or glucose metabolism.

Some observational studies reported lower 25OHD levels in pregnant women with GDM in comparison with normal pregnant women [169,170,171,172,173,174,175,176]. In Table 4, studies that evaluated serum 25OHD levels and the risk for GDM are shown.

**Table 4:** Observational studies on 25OHD serum levels and risk for GDM development.

Reference	Sample Size (GDM Cases *vs.* Controls)	Weeks of Gestation for Blood Sampling	25OHD Cut off	Risk for GDM Development (OR (95% CI))
[169]	20 GDM and 40 controls	At delivery	<50 nmol/L	30.78 (4.65–203.90)
[177]	68 GDM and 1,246 controls	26–28	<25 nmol/L	3.6 (1.7–7.8)
[171]	116 GDM and 219 controls	15–18	<73.5 nmol/L	2.21 (1.19–4.13)
[178]	200 GDM and 200 controls	26–28	<25 nmol/L	1.80 (1.209–2.678)
[172]	54 GDM and 111 controls	24–28	<37.5 nmol/L	2.66 (1.26–5.6)
			<50 nmol/L	2.02 (0.88–4.6)
[173]	57 GDM and 114 controls	16	<50 nmol/L	3.74 (1.47–9.50)
[170]	81 GDM and 226 controls	Between 2nd and 3rd trimester	<50 nmol/L	1.92 (0.89–4.17)

GDM = Gestational diabetes mellitus; OR = odds ratio; CI = confidence interval.

In a meta-analysis made by Poel and coworkers [179], the mean odds ratio calculated for GDM from 7 independent observational studies about 25OHD deficiency was 1.61 (95% CI 1.19–2.17). Similarly, a review on VD status and GDM risk concluded that maternal VD deficiency and insufficiency are associated with markers of altered glucose homeostasis [180].

Other studies correlated serum 25OHD levels with a significant inverse association with glucose metabolic response in women with GDM: higher serum 25OHD was found to be associated with at least one of the following parameters: lower fasting glucose, lower 2 h glucose (*post* oral glucose tolerance test), lower glycosylated hemoglobin, lower serum insulin, lower insulin resistance or lower homeostasis model of assessment of insulin resistance HOMA-IR [170,171,174,175,178,181,182,183,184].

In the future, the VD and lifestyle intervention (DALI) protocol could provide interesting information about VD supplementation and GDM risk. DALI is a multicenter European protocol in which the risk of GDM is being evaluated in 880 pregnant women divided into eight intervention groups considering physical activity, healthy eating habits and VD_3_ supplementation (1600 IU/day or placebo). Unfortunately, the results from this study are still unpublished [185]. To our knowledge, there is only one published double-blind randomized controlled clinical trial which supplemented VD_3_ on pregnant women with GDM [186]. In this study, pregnant women received two oral doses of 50,000 IU (baseline and day 21) or placebo capsules after 24 weeks of gestation. Women treated with VD_3_ had a significant increase in 25OHD serum levels and a significant decrease in fasting glucose, insulin serum and HOMA-IR, which supports positive glucose metabolic effects on mothers by VD_3_ supplementation. Similarly, Mozzafari and coworkers [187] administered a single intramuscular VD_3_ dose (300,000 IU) post-partum in 45 women with GDM. After three months of intervention, treated women presented significantly higher 25OHD levels and lower HOMA-IR than women with GDM in the control group.

Contradictorily to this background, there are some studies which concluded that there are no significant differences between VD status in women with GDM or controls [182,188,189], or conclude that VD deficiency is not a risk factor for GDM [190,191].

There is little consensus about the physiological mechanisms governing calcitriol and glucose metabolism connections. The classic mechanism known is that calcitriol can regulate intracellular calcium flux on β-pancreatic cells and therefore can modulate depolarization-stimulated insulin release [17]. However, there are recent evidences that include other VD-dependent mechanisms: (a) diminished inflammatory state in obesity and enhanced expression of genes involved in glucose and lipid metabolism like peroxisome proliferator-activated receptor gamma (PPARγ) or its coactivator (PGC1α) in peripheral blood mononuclear cells [192]; (b) weight reduction and muscle insulin receptor substrate 1 (IRS-1) upregulation [193]; (c) upregulation of adipocyte glucose transporter 4 (GLUT4) protein and its translocation to the cell surface [194]; and d) returning to normal liver activity of glucose metabolic enzymes hexokinase, fructose 1,6-bisphosphatase and glucose 6-phosphatase [195]. In GDM, these VD-modulated beneficial mechanisms could be altered, at least in part, because placentas from GDM mothers have higher CYP24A1 protein and gene expression which can derivate in lower calcitriol bioavailability [169].

### 7.4. Vitamin D and Low Birth Weight or Small for Gestational Age

Newborns small for gestational age (SGA) are defined as those with a birth weight for gestational age below to the 10th percentile in standard growth curves (<5th and 3rd percentiles are also used), whereas low birth weight is defined as newborn weight lower than 2500 g independently of gestational age [196,197]. These complications are associated with substantially higher rates of perinatal morbidity and mortality, including cerebral palsy, neonatal polycythemia, hyperbilirubinemia, and hypoglycemia [197].

The associations between serum maternal 25OHD levels and birth weight of SGA newborns have not been extensively studied. Epidemiological analyses support that black infants had lower birth weight than white infants, which suggests a possible role of VD [198]. However, there are still controversial opinions about lower VD levels failing to modulate fetal growth or if external parameters as maternal obesity, lower socioeconomic status or poor nutrition contribute to lower 25OHD levels and also to SGA and low birth weight development [199]. Herein, we present the major findings in this area. Few studies did not find a significant relation between birth weight for SGA proportion and 25OHD levels [190,200,201,202]; however, many observational studies did. A multicenter cohort study indicated that maternal 25OHD levels > 37.5 nmol/L are associated with higher birth weight infants in comparison to newborns from women with lesser than 37.5 nmol/L [203]. Another study indicates that pregnant women who delivered SGA infants had lower serum 25OHD levels at 11–13 gestational weeks [204]. Also, umbilical cord serum calcitriol concentrations were lower in SGA than in adequate weight for gestational age infants [205]. In this study, maternal 25OHD levels were also lower in the SGA group but did not reach statistical significance. Similarly, in a birth cohort study, women with 25OHD levels between 8.5 and 48 nmol/L were more likely to give birth to SGA offspring (OR 1.57, 95% CI 1.03–2.39) [206].

Dietary analysis of total VD_3_ intake was a significant predictor of infant birth weight adjusted for gestation [207], whereas milk or VD intake during pregnancy were significant independent predictors of birth weight [208]. However, it seems that placental weight is not related to 25OHD levels [203].

In a case-control study, Bodnar and colleagues [209] also observed that pregnant women with 25OHD deficiency (<37.5 nmol/L) had an increased significant risk of SGA development in offspring. Interestingly, this effect was more evident in white women (OR 7.5, 95% CI 1.8–31.9) in comparison to black women (OR 1.5, 95% CI 0.6–3.5). Unexpectedly, the women with 25OHD insufficiency (37.5–75 nmol/L) presented a lower risk of SGA than women with 25OHD sufficiency (>75 nmol/L) in both black and white women. The authors discuss that the potential mechanisms which can explain this U-shaped SGA risk remain uncertain, but the risk of other diseases such as allergic responses or atopic disorders present a similar pattern.

We only found one interventional study about VD and birth weight. In a partially randomized assay on pregnant women, the intervention with one single oral dose of 1500 μg VD_3_ (equal to 60,000 IU) or two doses of 3000 μg each (equal to 120,000 IU) in the 2nd and 3rd trimesters resulted in higher birth weight and length in comparison to newborns of mothers treated with usual care [210].

Interestingly, interventional studies with calcium and VD_3_ performed in pregnant adolescents showed that both nutrients positively influenced fetal bone growth *in utero*, and that even if both factors were needed for fetal bone health, one could partially compensate for the other [211]. This study suggests that special attention should be paid to pregnant adolescents in order to fulfill adequate VD and calcium requirements, since not only fetal but also maternal bone health may be at risk. This is supported by other studies showing that lactating adolescents lose more bone mineral density when suffering from VD deficiency or low calcium intake [212].

Interestingly, Morley and colleagues suggested that studies on maternal VD status and birth weight should consider neonatal VDR polymorphism, since differences in this feature could help explain why findings from different populations regarding maternal VD status and neonate birth weight have been inconsistent [213].

## 8. Vitamin D Expenditure and Homeodynamics: Considerations for VD Supplementation

### 8.1. Endogenous and Exogenous Factors Affecting the VD Status Equation

Considering the high risk of adverse events during pregnancy associated to VD deficiency, it is also important to analyze additional factors that may significantly modify the bioavailability of VD and its metabolites in our body. Indeed, the VD endocrine system is not in constant equilibrium; instead, it is under dynamic regulation and interaction with different factors. For example, the half-life of 25OHD is strongly influenced by DBP concentration, since 25OHD binds to DBP with high affinity [81,214]. Moreover, 25OHD is also affected by DBP genotype [214]. Indeed, the genetic variations that occur in DBP modify its binding affinity for VD metabolites, and the lesser affinity, a shorter half-life is expected. Similarly, genetic variations in CYP27B1, CYP24A1, CYP2R1 and the VDR differentially impact on VD metabolism and biological effects. Single nucleotide polymorphisms have been reported for each of these proteins and may be found as population-specific variants that result in modification of the final VD status. For reviews on this issue please see [215,216,217]. Considering this, and as previously suggested [215], it is feasible that optimal concentrations of 25OHD required to reduce disease outcomes may vary according to genotype. Other endogenous elements that may predict serum 25OHD half-life are factors known to affect 25OHD metabolism, such as PTH, plasma phosphate and albumin-adjusted calcium [218].

Besides the endogenous factors previously described, exogenous aspects affecting 25OHD plasma concentration include dietary intake, type of clothing and sunshine protection [219], lifestyle and geophysical conditions, this last one interpreted as UVB exposure. In this regard, a study performed with pregnant women in Germany showed that during the winter months, 98% of the maternal blood samples and 94% of the cord blood samples had 25OHD levels < 50 nmol/L, while in the summer months, only 49% of the women and 35% of the cord blood samples were vitamin D deficient [220]. Interestingly, in the same study, the authors found that a significant risk factor for maternal VD deficiency was physical inactivity (adjusted OR 2.67, 95% CI 1.06–6.69, *p* = 0.032), which might be related to less sun exposure. However, a sedentary lifestyle may also be associated with obesity, which has been found to be linked to VD deficiency. Indeed, obese subjects normally have lower basal 25OHD serum concentrations than lean individuals [221], which is possibly explained by the fact that VD is readily stored in adipose tissue due to its fat-soluble nature. In this manner, VD may be sequestered in the greater body pool of fat present in obese individuals, which is supported by previous studies showing that after equal whole-body irradiation or VD supplementation, the increase in serum VD was more than 50% lower in obese than in non-obese subjects [221]. While this occurs in body fat, the muscle cells protect 25OHD from degradation by binding it to actin fibers, in a megalin-dependent process [222]. This may explain why 25OHD concentrations are usually positively associated to muscle-related parameters such as lean body mass and exercise [223].

Unfortunately, during pregnancy, the toll of VD deficiency in obese mothers affects also their child’s VD status and health [224,225]. Many studies have shown an array of adverse health outcomes in the offspring of obese women; for example, lower maternal VD status may be linked to programmed differences in offspring fat mass [224,226]. Among the more important adverse effects of maternal VD deficiency upon their offspring, impaired fetal growth and bone development, altered growth and bone mass later in childhood, neonatal hypocalcemia or tetany and respiratory tract infections have been reported [227].

### 8.2. Dose Regimens and Vitamin D Supplementation in Pregnant Women

Despite the health advantages associated with a sufficient VD status during pregnancy, as described previously, at this time general consensus supporting a guideline for VD supplementation in pregnant population has not yet been reached. In the literature, a broad spectrum for dosage and periodicity in cholecalciferol supplementation schemes has been reported: 2000 IU, 4000 IU, 14,000 IU, 60,000 IU, 120,000 IU or 200,000 IU administered daily, weekly, monthly or in a single mega-dose. Herein, we resume the more recent randomized clinical trials on VD supplementation in pregnancy. Table 5 includes doses and frequency for supplementation together with the outcome as percentage of women who achieved VD sufficiency at the end of intervention and those who developed hypercalcemia.

Based on these data, it seems that 4000 IU given daily results in the highest proportion of pregnant women reaching VD sufficiency without developing hypercalcemia. An exception to this observation is the study by Hossain *et al.* [228], in which only 15% women reached sufficiency under this regimen. Regarding this, we should mention that the group of Pakistani women included in the Hossain study were severely VD deficient (<25 nmol/L) and were an ethnic group in which particular genetic variants might be affecting VD metabolism, which remains to be further studied. Moreover, the cases of hypercalcemia were comparable in both control and supplemented groups, while hypercalcemia persisted despite VD deficiency, suggesting independence of the pharmacological intervention. On the other hand, it should be noted that in the study by Wagner *et al.* [229], the percentage of women considered to attain sufficiency might be underestimated, since their cut-off value was 25OHD serum levels > 100 nmol/L. As in the case of 4000 IU given daily, the weekly regimen of 50,000 IU seems to be also a good therapeutic strategy, since 100% women reached sufficiency without hypercalcemia. However, more studies are needed to confirm these findings.

In contrast, monthly and unique dose regimens do not seem to adequately fulfill VD sufficiency. It is noteworthy that under the single-dose regimen, some authors included in their analyses serum levels below 75 nmol/L (identified with * and ** in Table 5), which under the Endocrine Society parameters is still considered deficiency. Indeed, in the study by Sahu *et al.* [230], which considered a cut-off of 75 nmol/L, only 34.2% of women reached sufficiency with the highest dose, which is very low. Similarly, in the study by Yu *et al.* [231], the observed proportion of 93% in the supplemented group might be misleading, since this number includes women with serum 25OHD levels > 25 nmol/L.

The recent evidence discussed in Section 8.1 may help us to understand the differences in VD expenditure and homeodynamics in order to reach a general consensus on VD supplementation in a tailored manner. We believe, that at this moment, the ideal scheme for VD supplementation will depend on particular endogenous and exogenous factors, on the available formulations of VD (*i.e.*, in Mexico only tablets with 400 IU are available), and on the personal VD metabolism, which should be monitored periodically by serum 25OHD analyses. By taking all these considerations into account, and under medical counseling and supervision, every woman may modulate their VD levels for acquiring sufficiency and avoiding possible toxicity that could lead to hypercalcemia.

**Table 5:** Cholecalciferol supplementation and VD status in randomized clinical trials in healthy pregnant women.

Reference	Sample Size	Period of Supplementation	Cholecalciferol Supplemented (IU)	% Women with Serum 25OHD > 75 nmol/L at Delivery (No Asterisk)	Hypercalcemia
***Unique Dose***					
[210]	97	2nd trimester	60,000	27% **	No evaluated
		Two doses at 2nd and 3rd trimester	120,000	62.5% **	
[230]	84	No supplementation	-	7%	No evaluated
		5th month	60,000	5.7%	
		Two doses at 5th and 7th month	120,000	34.2%	
[231]	180	No supplementation	-	60% *	No indicated
		27 week	200,000	93% *	
***Daily***					
[232]	228	27 weeks to term	0	50%	No
			1000	89%	
			2000	91%	
[228]	175	Less than 20 weeks to term	0	1%	3 cases
			4000	15%	9 cases
[233]	162 deficient women	12–16 weeks to term	400	9.5%	No
			2000	24.4%	
			4000	65.1%	
[229]	257	12–16 weeks to term	2000	37.4% ***	No
			4000	46.2% ***	
[234]	350	12–16 weeks to term	400	50%	No
			2000	70.8%	
			4000	82%	
[231]	180	27 weeks to term	800	86% *	No indicated
***Weekly***					
[235]	109 deficient women	26–28 weeks to term (8 weeks)	400	3.70%	No
			50,000	100%	
[236]	28	26–28 weeks to term	Basement 70,000 + 35,000 weekly	90%	No
			14,000 weekly	56%	
***Monthly***					
[237]	51 deficient women	From 2nd month to term	50,000	35% ****	No evaluated
			100,000	59% ****	

* 25OHD serum > 25 nmol/L; ** 25OHD serum > 50 nmol/L; *** 25OHD serum > 100 nmol/L; **** in cord blood.

## 9. Methods of Serum VD Measurement

Since four decades ago, numerous analytical methods have been developed for 25OHD measurement, including competitive protein binding assay, enzyme-linked immunoassay (ELISA), radioimmunoassay (RIA), chemiluminescence assay, gas chromatography-mass spectrometry (GC-MS), high performance liquid chromatography (HPLC) and, more recently, liquid chromatography coupled with mass spectrometry (LC-MS) or tandem mass spectrometry (LC-MS/MS). A good review of accuracy, sensibility and technical description of these methods was made by Hollis [238].

LC-MS/MS is the most promising technique for VD analysis since it is a highly specific, reliable, reproducible and robust method and is considered the new gold standard for 25OHD quantification [239,240]. Despite its major field of application being research, LC-MS/MS technology is also currently being applied in clinical laboratories [241]. In addition, LC-MS/MS offers the possibility for quantifying other metabolites of VD in serum samples, such as 25OHD_2_, 25OHD_3_, 3-epi-25OHD_2_, 3-epi-25OHD_3_, 24R,25 dihydroxyvitamin D_3_ [242,243] and 25OHD_3_ 3-sulfate [244]. Other metabolites as VD_2_, VD_3_, 1α,25(OH)_2_D_2_ and 1α,25(OH)_2_D_3_ may also be detected and discriminated.

One limitation of this technique is the interference with 3-epi 25(OH)D_3_ that can lead to 25OHD overestimation. This could be a problem especially in pediatric samples which are known to have significant amounts of this epimer. Recently, van den Ouweland and colleagues developed a LC-MS/MS method which eliminates this limitation [245].

Interestingly, new technology developed to be used in our daily life will allow us to measure 25OHD easily at home. The proposed system is a gold-nanoparticle-immunoassay developed at Cornell University, adapted to a device that couples to smartphones allowing them to calculate in a small drop of blood 25OHD serum concentrations with 10 nM sensitivity [246].

In the aftermath of what is discussed in Section 5 and Section 8.1, it seems that 25OHD should not be exclusively considered for the assessment of VD, but rather, the equation for VD status may also consider DBP levels. Powe and coworkers [247] recently suggested that free 25OHD (25OHD minus DBP) is a better indicator for VD status since free 25OHD offers a strong correlation with PTH than total 25OHD. However, this article has been criticized by other authors [248]. Indeed, as previously discussed, Weintraub [248] pointed out that the 25OHD/DBP complex is necessary for the endocytosis by megalin/cubilin in the kidney, so 25OHD bound to DBP may be the real substrate mediating final calcitriol biosynthesis. Nevertheless, this would only apply to those cells expressing megalin/cubilin, and definitive further studies are needed in order to clarify the participation of other mechanisms of 25OHD storage and internalization into the cell.

## 10. Final Considerations

Maintaining adequate VD serum concentrations within the recommended levels is mandatory during pregnancy, since it is involved in many important biological processes, including fetal programming and development. Indeed, the benefits of maintaining adequate VD serum levels are not circumscribed to the mother, but also to the offspring. It is noteworthy mentioning that epidemiological studies have shown controversial results about the benefits of prescribing VD supplements to prevent adverse pregnancy outcomes associated with maternal deficiency. We believe that the controversy may be explained because the biological phenomena can be affected not only by serum concentrations, but also by many factors, such as racial, climatological, or genetic reasons; nutritional status; lifestyle; physical activity; or health status during pregnancy. On the other hand, the few studies that do not corroborate benefits lack sustained clinical evidence of real risks due to VD supplementation. Conversely, systematic reviews and meta-analyses demonstrate a strong association between VD adequate levels and health benefits. Regarding this controversy, recently, two debated articles concluded that the proposed adverse health outcomes related to VD deficiency might be, in fact, the result of reverse causation, understanding by this that low VD levels are a consequence of ill health rather than a cause, and that the evidence does not really support VD supplementation for prevention of disease [249,250,251]. In response to these articles, Gillie, with straightforward arguments, demonstrated that the aforementioned articles had made a type 2 statistical error, and that VD deficiency, especially at critical times such as pregnancy and early childhood, could derive in serious health harm [252]. An example of the arguments exposed by Gillie is rickets, a disease characterized by bone deformation in children caused by VD deficiency. This illness may be corrected by adequate VD supplementation during childhood, but the alterations in bones cannot be reversed by this intervention once adulthood is reached. Another example discussed by Gillie is diabetes type 1, which occurs in children and is thought to be caused by VD deficiency in the womb, causing irreversible changes to biochemistry, immune status or organ structure.

A final consideration is the fact that VD supplementation is useful to prevent adverse pregnancy outcomes, but it might not always be necessary, especially when lifestyle recommendations are good enough to prevent them. In order to take the adequate decision about VD supplementation, every clinical individual situation must be analyzed and placed in the correct balance of risk and benefit before prescribing VD supplementation. However, when controversies about clinical decisions are involved, scientists must avoid creating medical barriers about the use of preventive strategies in medicine.

## 11. Conclusions

Although the importance of VD in the regulation of calcium homeostasis in pregnant women is well established, there is now increasing evidence that calcitriol is also important for the prevention of several adverse scenarios that could potentially threaten pregnancy such as infection and preeclampsia. Even though more interventional and basic studies are needed in order to understand the role of VD in pregnancy health and disease, through the information resumed herein it is clear that many of the beneficial effects of calcitriol during gestation involve its immunomodulatory properties as well as its capacity to regulate hormonogenesis. Despite the protective role of VD in pregnancy outcomes and that several epidemiological studies have documented highly prevalent gestational hypovitaminosis D around the world, routine VD screening is still not mandatory and not enough interventional studies have been undertaken to achieve a consensus for VD supplementation in pregnant women, highlighting the need for further studies and establishment of screening guidelines during pregnancy. Given that the human placenta expresses CYP27B1, which catalyzes the local synthesis of calcitriol, the supplementation with VD during pregnancy might be an accessible and safe way to reduce the incidence of some adverse events associated with mother and baby morbidity-mortality, such as PE, GDM, PROM and infections; while, at the same time, both the mother and the child will profit from the physiological benefits of calcitriol. Notably, adequate sun exposure, a VD-rich diet and physical activity should always be considered as the first recommendation, while supplementation with cholecalciferol may be advised for persistent VD deficient women.
